# Medical Politics in New York

**Published:** 1852-12

**Authors:** 


*Medical Politics in New York.* — We copy the following paragraph, with the above heading, from the Western Medico-Chirurgical Journal. We do not see the *Scalpel,* but the reference made to the two other journals named, we can appreciate, as both are among our exchanges. In common, we presume, with almost every member of the profession who receives either of these medical publications, we have read with emotions of regret the acerbity of tone which has characterized many of the editorial articles for the last few months. Whether the sweeping charge against the profession of the city of New York, contained in the first part of the following paragraph be correct, we will not assume to say. In the concluding remarks of the editor we candidly concur: "There seems to be no city in the Union where professional bickerings and animosities have gone so far as in the great metropolis. Every man's hand seems to be against his neighbor, and even the oldest and most experienced physicians — whose claims to respect are everywhere else acknowledged — seem not to escape the general strife. We know but little of the merits of the controversy carried on between the New York Gazette and Scalpel on the one hand, and the Northern Lancet on the other; but if the parties were content to select some medical topic for discussion, and then enter the arena animated by a generous spirit of emulation, medical science wonld not suffer thereby."

